# Supplementary material for: Fibromodulin selectively accelerates myofibroblast apoptosis in cutaneous wounds by enhancing interleukin 1β signaling
Source: Nat Commun. 2025 Apr 12;16:3499. doi: 10.1038/s41467-025-58906-z (PMC11993684; doi:10.1038/s41467-025-58906-z)
Supplement: Supplementary file 1 — Supplementary Information [file 41467_2025_58906_MOESM1_ESM.pdf]

Supplementary Information

Supplementary Table

Supplementary Table 1. Primary adult human dermal fibroblasts and skin tissues used in the current study.

Primary adult human dermal fibroblasts				
**Cell ID**	**Gender**	**Catalogue #**	**Lot. /batch #**	**Source**
KB-AA35 (KEL FIB)	Female	CRL-1762	70015321	ATCC
FB-AA36 (HDFa)	Female	PCS-201-012	80616177	
FB-C31 (NHDF-Ad)	Female	CC-2511	109944	Lonza
FB-C51 (NHDF-Ad)	Male	CC-2511	293971	
**Human skin tissues**				
**Donor reference ID**	**Gender**	**Skin type**	**Location**	**Source**
1914138	Female	Normal	Abdomen	NDRI
		Keloid	Abdomen	
		Hypertrophic scar	Left Shoulder	
2106181	Female	Normal	Right Knee	
		Keloid	Right Knee	
1914476	Male	Normal	Abdomen	
		Hypertrophic scar	Abdomen	
1914478	Female	Normal	Left Elbow	
		Hypertrophic scar	Left Elbow	

Supplementary Figures

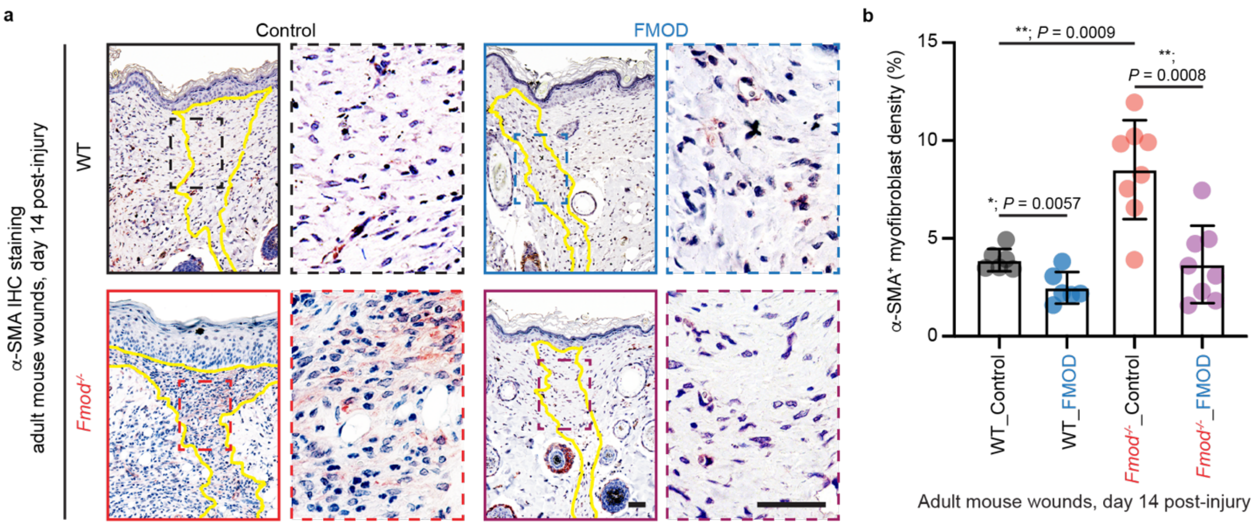

Supplementary Fig. 1. Fibromodulin (FMOD) accelerates myofibroblast clearance in mouse wounds.

a. Representative images of sections stained with α-smooth muscle actin (α-SMA) by immunohistochemical (IHC) staining from adult wildtype (WT) and *Fmod*$^{-/-}$ mouse wounds at day 14 post-injury. All treatments were administrated at the time of surgery. Yellow lines outline the scar area. Dashed boxes in the lower magnification images represent the region of interest shown in the higher magnification image to the immediate right of each lower magnification image. **b**. Quantification of α-SMA^{+} myofibroblast density in adult mouse wounds from **a**. The number of α-SMA positively stained cells and nuclei across the entire wound area was counted under a microscope from two centrally bisected sections of each wound sample. The ratio of α-SMA^{+} cells to the total number of cells (indicated by the number of nuclei) was then calculated to quantify the density of α-SMA^{+} myofibroblasts. Scale bars, 50 μm. Data presented as mean ± standard deviation (s.d.) overlaying all the data points. *N* = 6 (WT) or 8 (*Fmod*$^{-/-}$) mice, respectively; *P* values were determined by two-tailed unpaired *t*-tests (**b**). *, *P*

< 0.05; **, P < 0.005. Source data are provided as a Source Data file.

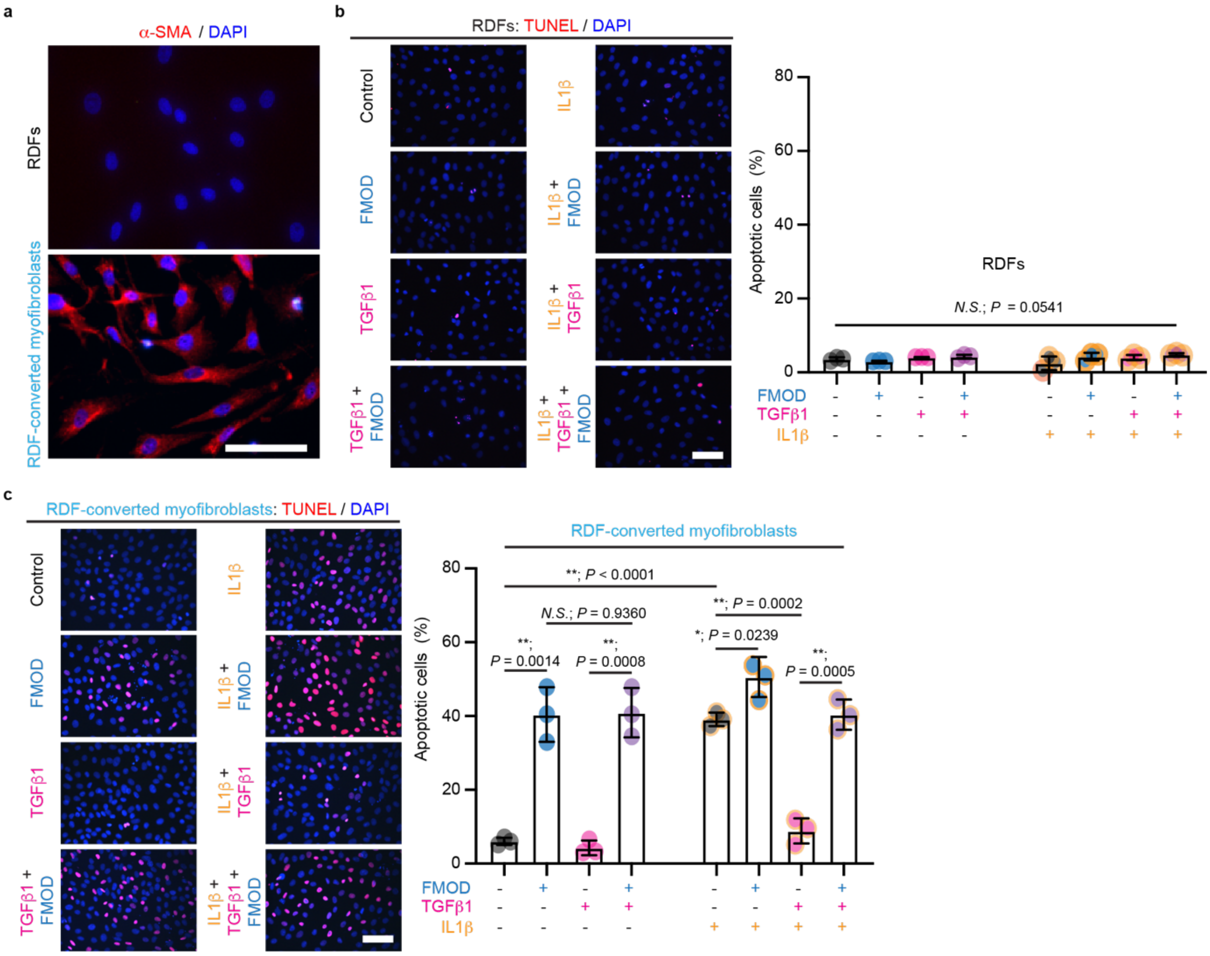

Supplementary Fig. 2. Fibromodulin (FMOD) induces apoptosis of rat dermal fibroblast (RDF)-converted myofibroblasts.

All cells were subjected to serum starvation prior to treatment. **a**. Representative images of RDFs stained with α-smooth muscle actin (α-SMA) by immunofluorescence before and after 2.5 ng/mL transforming growth factor β1 (TGFβ1) treatment for 4 days. **b**. Representative images and quantification of RDFs stained with terminal deoxynucleotidyl transferase dUTP nick end labeling (TUNEL) assay. **c**. Representative images and quantification of RDF-converted myofibroblasts with TUNEL staining. The number of TUNEL-positively stained cells among 1,000 cells (determined by DAPI-stained nuclei) was counted under a microscope for each cell culture to determine the percentage of apoptotic cells. Scale bars, 50 μm. Data presented as mean ± standard deviation (s.d.) overlaying all the data points. *N* = 3

different cell cultures; *P* values were determined by one-way ANOVA (**b**) or two-tailed unpaired *t*-tests (**c**). *N.S.*, not significant, $P > 0.05$; *, $P < 0.05$; **, $P < 0.005$. Source data are provided as a Source Data file.

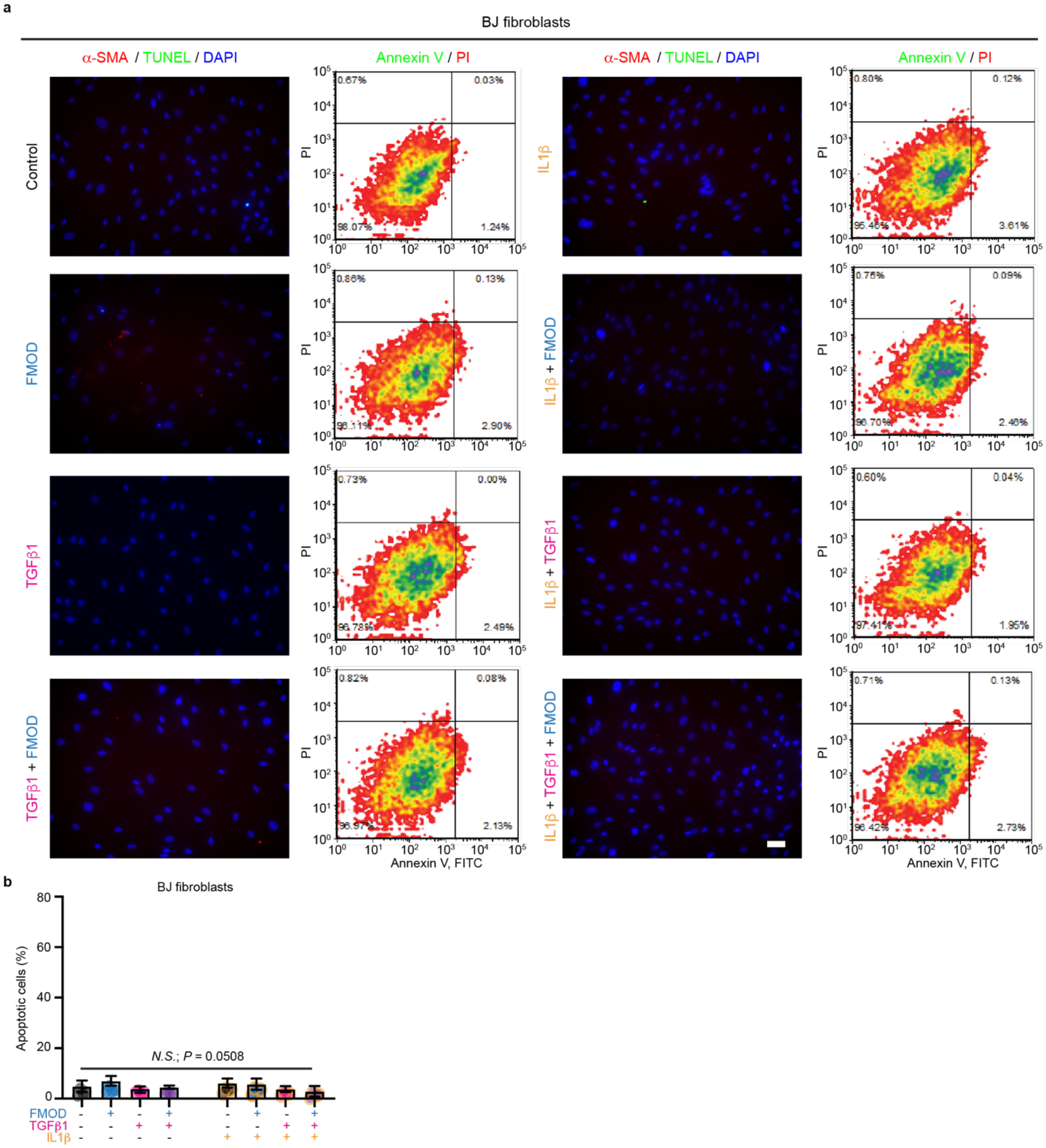

Supplementary Fig. 3. Fibromodulin (FMOD) does not induce human BJ fibroblast apoptosis.

All cells were subjected to serum starvation prior to treatment. **a**. Representative images of BJ fibroblasts with terminal deoxynucleotidyl transferase dUTP nick end labeling (TUNEL) staining, accompanied by the respective flow cytometry plots stained with Annexin V-fluorescein isothiocyanate (FITC) and propidium iodide (PI) staining.

b. Quantification of apoptotic BJ-myofibroblasts by flow cytometry from **a**. Scale bars, 25 μm. Data presented as mean ± s.d. overlaying all the data points. *N* = 3 (for TUNEL staining) or 4 (for flow cytometry) biological replicates; *P* value was determined by one-way ANOVA (**b**). *N.S.*, not significant, $P > 0.05$. Source data are provided as a Source Data file.

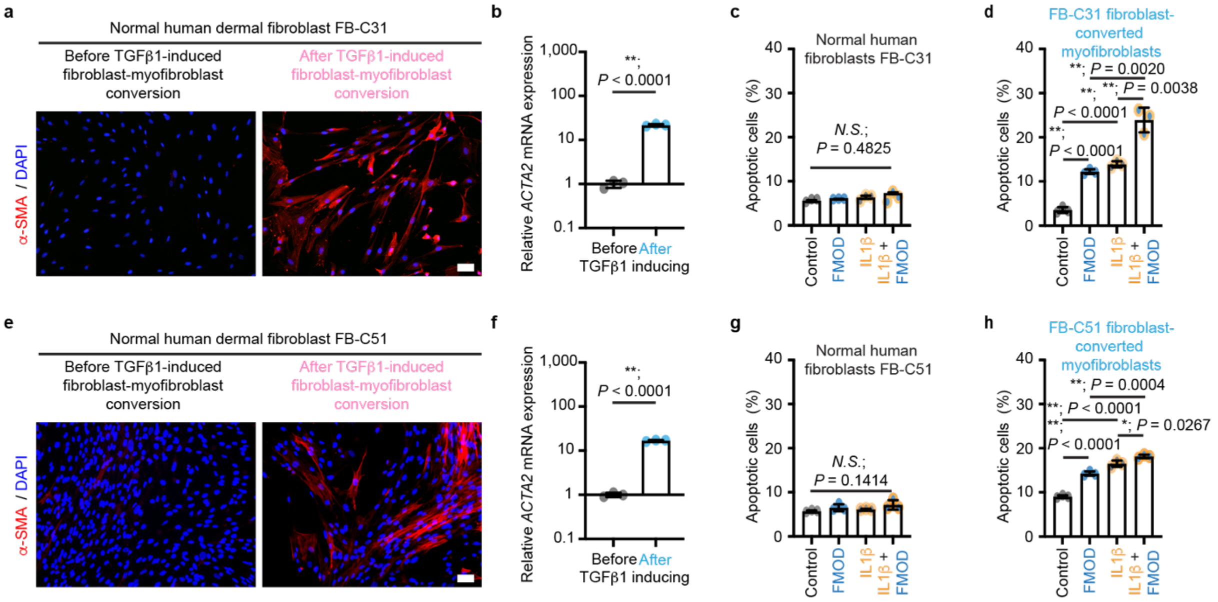

Supplementary Fig. 4. Fibromodulin (FMOD) induces the apoptosis of the primary normal human dermal fibroblast (NHDF) FB-C31- and FB-C51-converted myofibroblasts.

All cells were subjected to serum starvation prior to treatment. **a**. Representative images of the primary NHDF FB-C31 (derived from a 31-year-old female Caucasian donor; Supplementary Table 1) with α-smooth muscle actin (α-SMA) immunofluorescence staining before and after transforming growth factor (TGF)β1-induced fibroblast-myofibroblast conversion. **b**. Expression of *ACTA2* in FB-C31 fibroblasts before and after fibroblast-myofibroblast conversion. Gene expression data were normalized to the *ACTA2* level of FB-C31 fibroblasts before fibroblast-myofibroblast conversion. **c**. Quantification of FB-C31 fibroblast apoptosis by flow cytometry with Annexin V-FITC and PI staining. **d**. Quantification of FB-C31 fibroblast-converted myofibroblast apoptosis by flow cytometry with Annexin V-FITC and PI staining. **e**. Representative images of the primary NHDF FB-C51 (derived from a 51-year-old male Caucasian donor; Supplementary Table 1) with α-SMA immunofluorescence staining before and after TGFβ1-induced fibroblast-

myofibroblast conversion. **f**. Expression of *ACTA2* in FB-C51 fibroblasts before and after fibroblast-myofibroblast conversion. Gene expression data were normalized to the *ACTA2* level of FB-C51 fibroblasts before fibroblast-myofibroblast conversion. **g**. Quantification of FB-51 fibroblast apoptosis by flow cytometry with Annexin V-FITC and PI staining. **h**. Quantification of FB-C51 fibroblast-converted myofibroblast apoptosis by flow cytometry with Annexin V-FITC and PI staining. Scale bars, 25 μm. Data presented as mean ± s.d. overlaying all the data points. $N = 3$ biological replicates; P values were determined by two-tailed unpaired *t*-tests (**b**, **d**, **f**, and **h**) or one-way ANOVA (**c** and **g**), respectively. *N.S.*, not significant, $P > 0.05$; *, $P < 0.05$; **, $P < 0.005$. Source data are provided as a Source Data file.

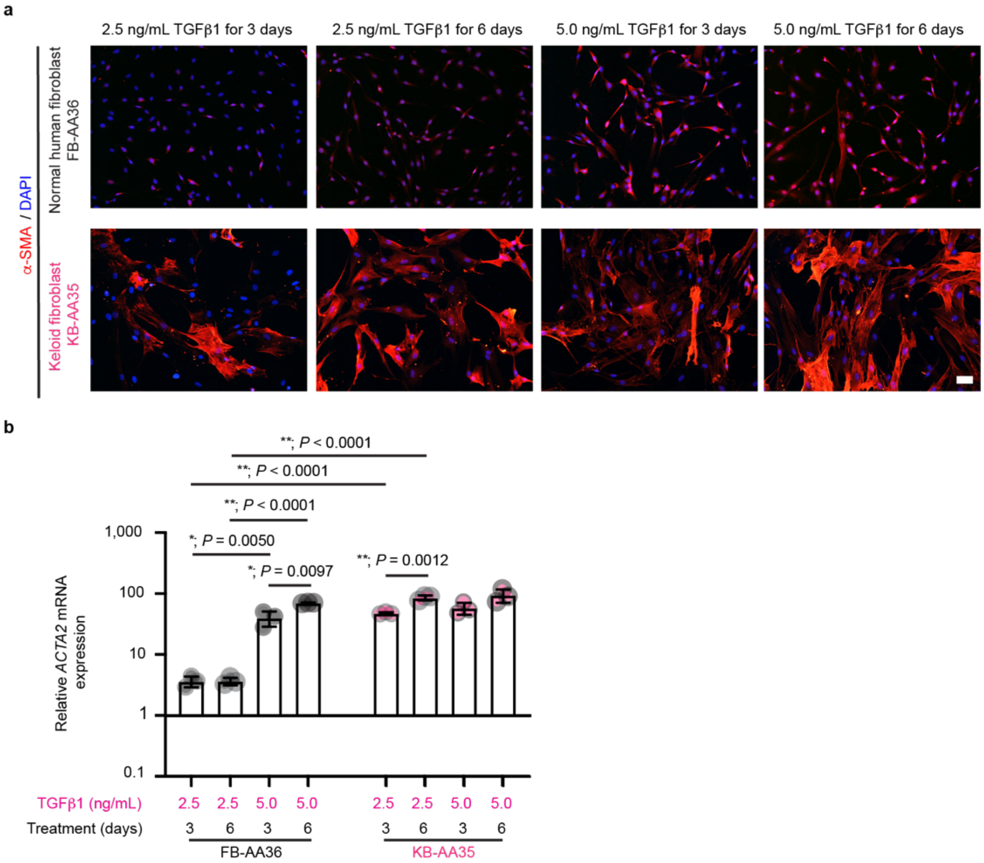

Supplementary Fig. 5. Keloid fibroblasts display a more rapid and severe α-smooth muscle actin (α-SMA) expression response to transforming growth factor (TGF)β1 than normal human dermal fibroblasts.

All cells were subjected to serum starvation prior to treatment. **a**. Representative images of FB-AA36 and KB-AA35 fibroblasts with α-SMA immunofluorescence staining after TGFβ1-induced fibroblast-myofibroblast conversion. **b**. Expression of *ACTA2* in FB-AA36 and KB-AA35 fibroblasts after TGFβ1-induced fibroblast-myofibroblast conversion. Data were normalized to the *ACTA2* level of the respective fibroblasts before fibroblast-myofibroblast conversion. Scale bars, 25 μm. Data presented as mean ± s.d. overlaying all the data points. $N = 3$ biological replicates; P values were determined by two-tailed unpaired t-tests (**b**). *, $P < 0.05$; **, $P < 0.005$. Source data are provided as a Source Data file.

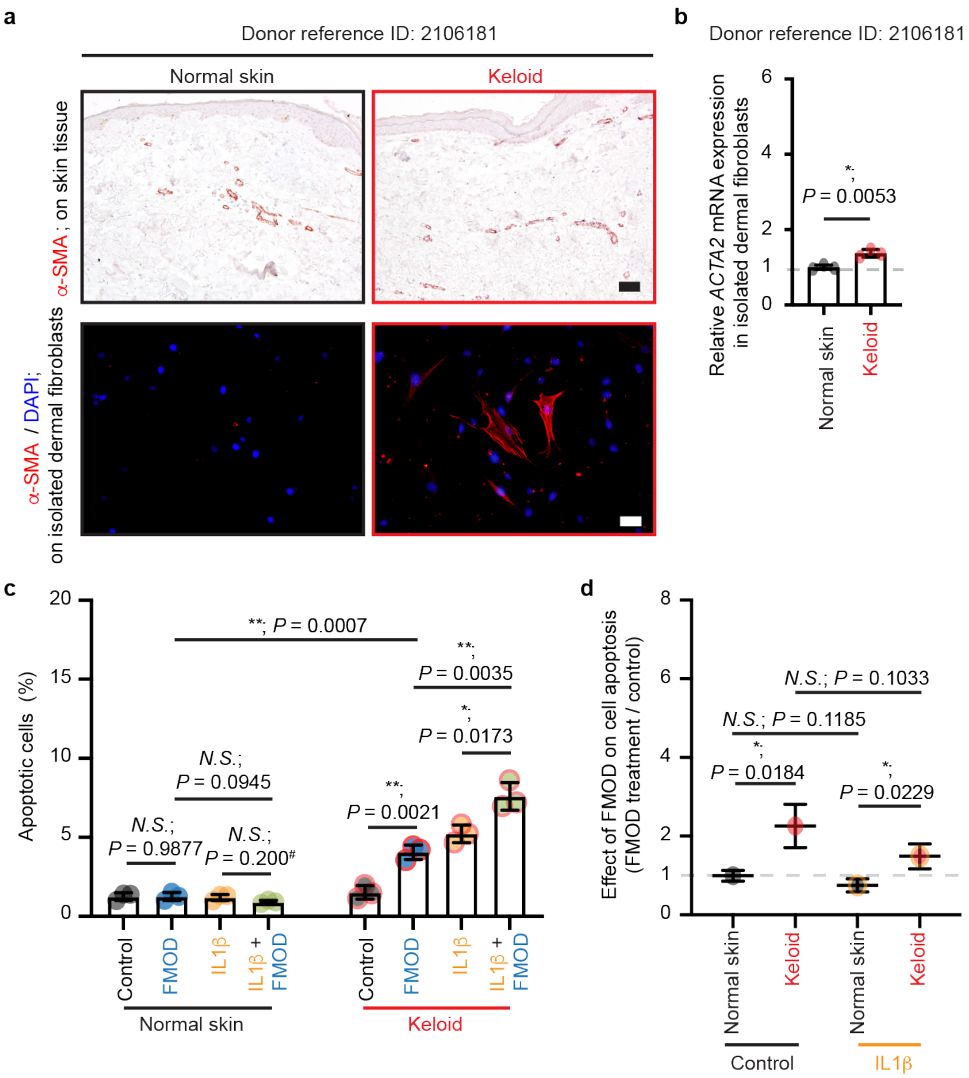

Supplementary Fig. 6. Fibromodulin (FMOD) induces the apoptosis of keloid-derived dermal fibroblasts of donor 2106181.

All cells were subjected to serum starvation prior to treatment. **a**. Representative images of normal skin and keloid tissues collected from the right knee of the donor with the NDRI donor reference ID 2106181 (Supplementary Table 1) with α-smooth muscle actin (α-SMA) immunohistochemical staining (upper) and the representative images of dermal fibroblasts isolated from these tissues with α-SMA immunofluorescence staining (lower). **b**. Expression of *ACTA2* in dermal fibroblasts isolated from the tissues described in **a**. Data were normalized to the *ACTA2* level of fibroblasts isolated for the normal skin. **c**. Quantification of apoptosis of dermal

fibroblasts isolated from tissues described in **a**. **d**. Impacts of FMOD on cell apoptosis determined from **c**. Scale bars, 50 μm (black) or 25 μm (white). Data presented as mean ± s.d. overlaying all the data points. *N* = 3 biological replicates; *P* values were determined by two-tailed unpaired *t*-tests (**b-d**), except the one with marker # (**c**, in which data of IL1β-treated cells derived from normal skin tissues did not meet the criteria for normal distribution test and thus was treated as non-parametric) where a Mann-Whitney *U* test determined *P* value. *N.S.*, not significant, $P > 0.05$; *, $P < 0.05$; **, $P < 0.005$. Source data are provided as a Source Data file.

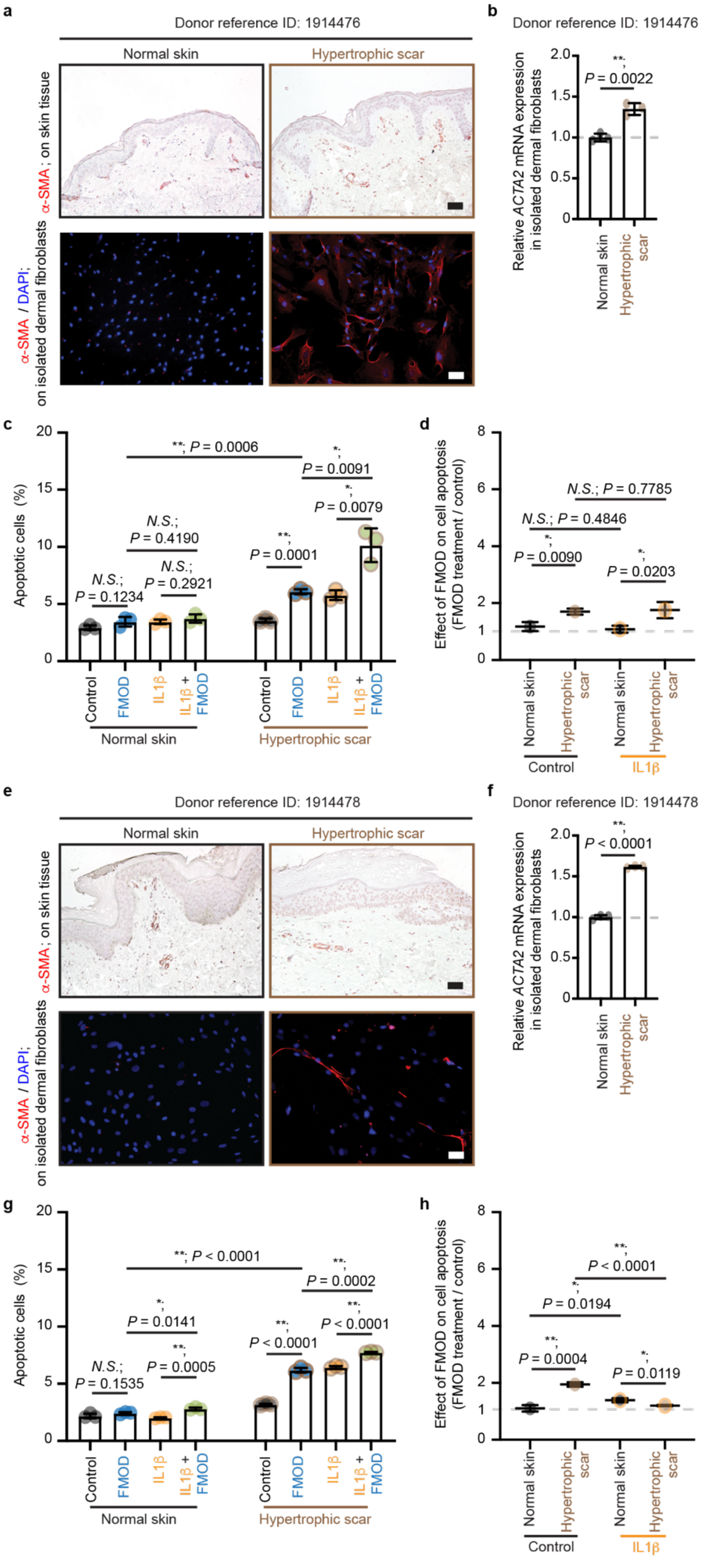

Supplementary Fig. 7. Fibromodulin (FMOD) induces the apoptosis of the

hypertrophic scar-derived dermal fibroblasts.

All cells were subjected to serum starvation prior to treatment. **a**. Representative images of normal skin and hypertrophic scar tissues collected from the abdomen of the donor with the NDRI donor reference ID 1914476 (Supplementary Table 1) with α-smooth muscle actin (α-SMA) immunohistochemical staining (upper) and the representative images of dermal fibroblasts isolated from these tissues with α-SMA immunofluorescence staining (lower). **b**. Expression of *ACTA2* in dermal fibroblasts isolated from the tissues described in **a**. Data were normalized to the *ACTA2* level of fibroblasts isolated for the normal skin. **c**. Quantification of apoptosis of dermal fibroblasts isolated from the tissues described in **a**. **d**. Impacts of FMOD on cell apoptosis determined from **c**. **e**. Representative images of normal skin and hypertrophic scar tissues collected from the left elbow of the donor with the NDRI donor reference ID 1914478 (Supplementary Table 1) with α-SMA immunohistochemical staining (upper) and the representative images of dermal fibroblasts isolated from these tissues with α-SMA immunofluorescence staining (lower). **f**. Expression of *ACTA2* in dermal fibroblasts isolated from the tissues described in **e**. Data were normalized to the *ACTA2* level of fibroblasts isolated for the normal skin. **g**. Quantification of apoptosis of dermal fibroblasts isolated from the tissues described in **e**. **h**. Impacts of FMOD on cell apoptosis determined from **g**. Scale bars, 50 μm (black) or 25 μm (white). Data presented as mean ± s.d. overlaying all the data points. $N = 3$ biological replicates; P values were determined by two-tailed unpaired *t*-tests (**b-d** and **f-h**). *N.S.*, not significant, $P > 0.05$; *, $P < 0.05$; **, $P < 0.005$. Source data are provided as a Source Data file.

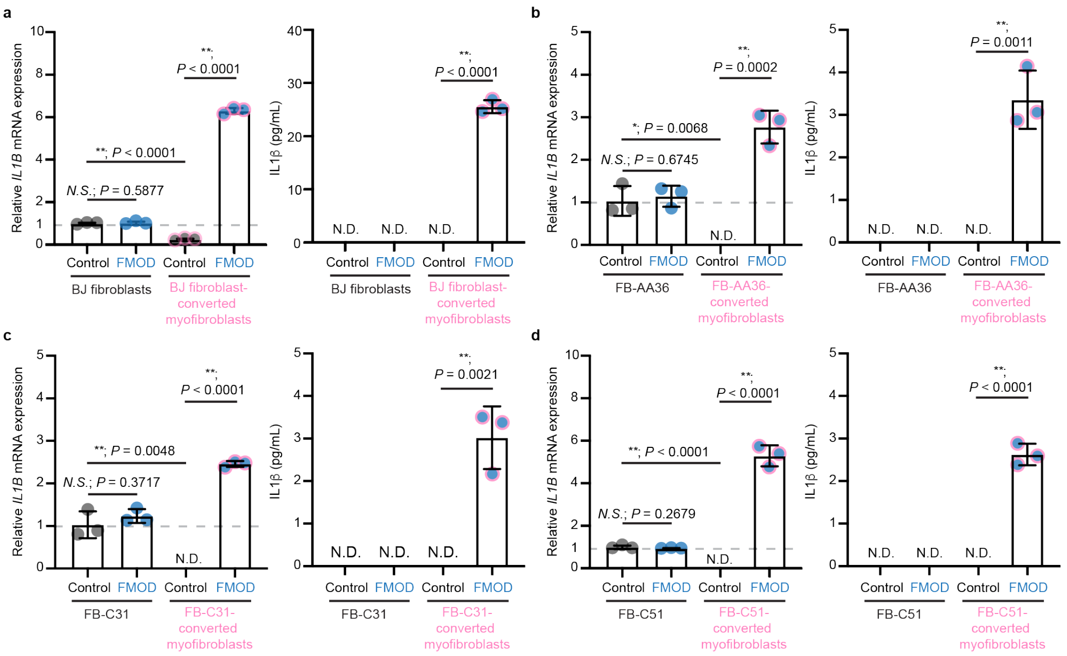

Supplementary Fig. 8. Fibromodulin (FMOD) selectively induces interleukin (IL)1β expression in the converted human myofibroblasts but not their fibroblast precursors.

All cells were subjected to serum starvation prior to treatment. **a-d**. Expression of *IL1B* and production of active IL1β of neonatal human foreskin fibroblast BJ (**a**), adult NHDF FB-AA36 (**b**), FB-C31 (**c**), and FB-C51 (**d**), and their myofibroblast derivatives. Gene expression data were normalized to the *IL1B* level of the respective unconverted fibroblasts without FMOD treatment. Data presented as mean ± s.d. overlaying all the data points. N = 3 biological replicates; P values were determined by two-tailed unpaired t-tests (**a-d**). N.D., not detectable; *N.S.*, not significant, $P > 0.05$; *, $P < 0.05$; **, $P < 0.005$. Source data are provided as a Source Data file.

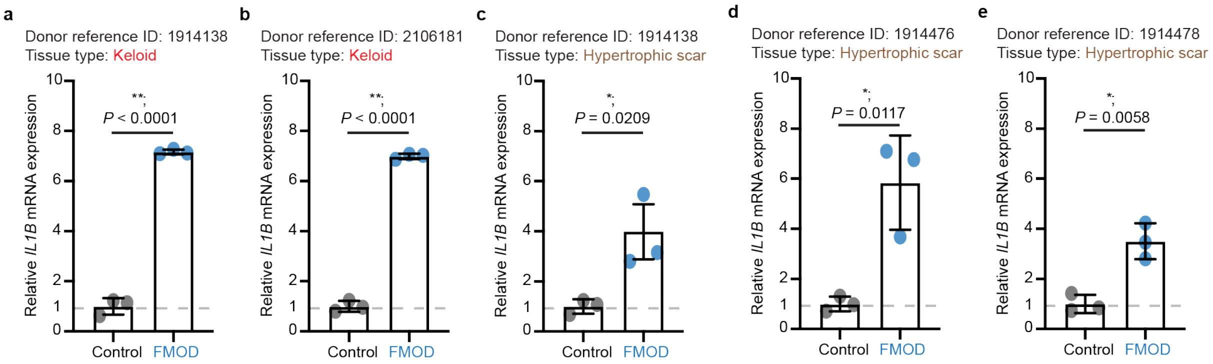

Supplementary Fig. 9. Fibromodulin (FMOD) upregulates keloid- and hypertrophic scar-derived fibroblast interleukin (IL)1β expression.

All cells were subjected to serum starvation prior to treatment. **a-e**. Expression of *ACTA2* in dermal fibroblasts isolated of keloid (**a**, **b**), and hypertrophic scar (**c**, **d**, **e**) tissues of donor with NDRI donor reference ID 1914138 (**a**, **c**), 2106181 (**b**), 1914476 (**d**), and 1914478 (**e**), respectively. Data were normalized to the *ACTA2* level of the respective control cells. Data presented as mean ± s.d. overlaying all the data points. *N* = 3 biological replicates; *P* values were determined by two-tailed unpaired *t*-tests (**a-e**). *, *P* < 0.05; **, *P* < 0.005. Source data are provided as a Source Data file.

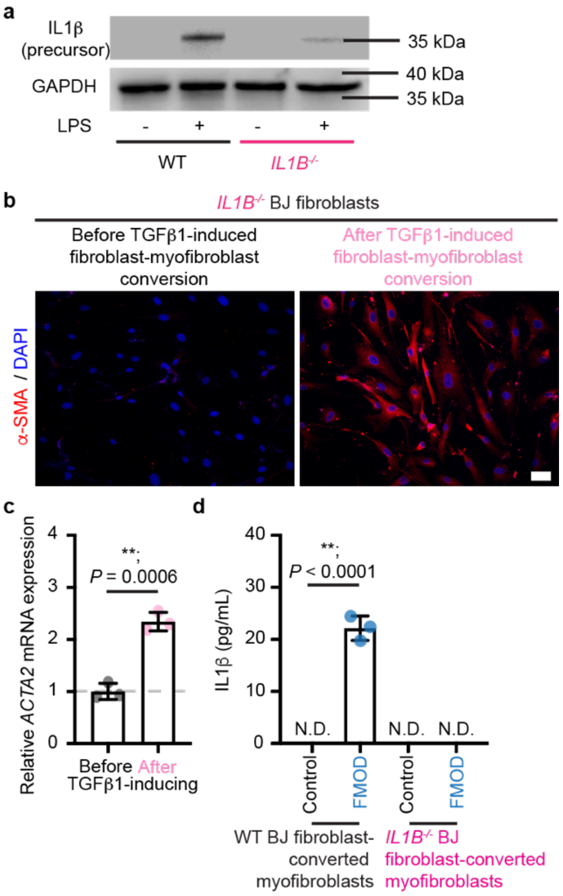

Supplementary Fig. 10. *Interleukin (IL)1B-knockout* does not prohibit fibroblast-to-myofibroblast conversion.

All cells were subjected to serum starvation prior to treatment. **a**. Representative western blotting image of IL1β production from wildtype (WT) and *IL1B-knockout* (*IL1B*$^{-/-}$) BJ fibroblasts with or without lipopolysaccharide (LPS) stimulation. Glyceraldehyde-3-phosphate dehydrogenase (GAPDH) was used as the protein loading control. **b**. Representative images of *IL1B*$^{-/-}$ BJ fibroblasts with α-smooth muscle actin (α-SMA) immunofluorescence staining before and after transforming growth factor (TGF)β1-induced fibroblast-myofibroblast conversion. **c**. Expression of *ACTA2* in *IL1B*$^{-/-}$ BJ fibroblasts before and after TGFβ1-induced fibroblast-myofibroblast conversion. Data were normalized to the *ACTA2* level before fibroblast-myofibroblast conversion. **d**. IL1β secretion by WT and *IL1B*$^{-/-}$ BJ fibroblast-converted

myofibroblasts with or without FMOD treatment. Scale bar, 25 μm. Data presented as mean ± s.d. overlaying all the data points. *N* = 3 biological replicates; *P* values were determined by two-tailed unpaired *t*-tests (**c-d**). N.D., not detectable; **, $P < 0.005$. Source data are provided as a Source Data file.

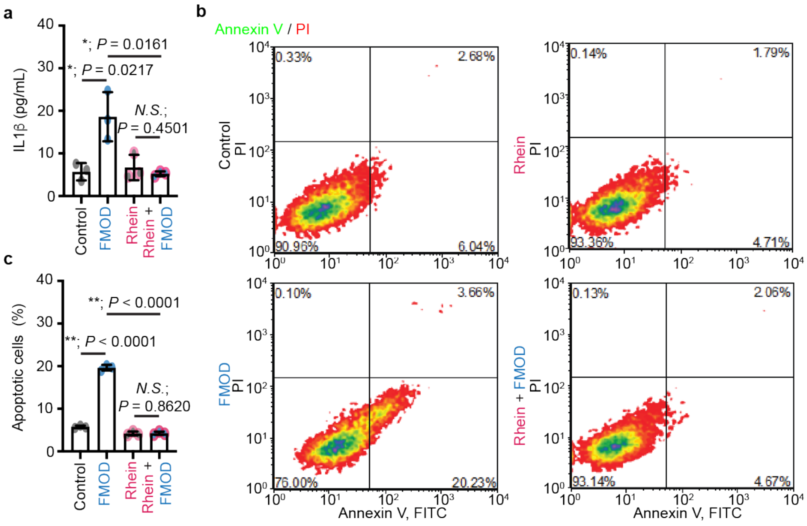

Supplementary Fig. 11. Rhein impairs the fibromodulin (FMOD)-induced apoptosis of BJ-myofibroblasts.

All cells were subjected to serum starvation prior to treatment. **a**. Active interleukin (IL)1β production in BJ-myofibroblasts with or without rhein treatment. **b**. Representative plots of BJ-myofibroblasts with or without rhein treatment stained with Annexin V-FITC and PI staining. **c**. Quantification of BJ-myofibroblast apoptosis from **b**. Data presented as mean ± s.d. overlaying all the data points. N = 3 biological replicates; P values were determined by two-tailed unpaired t-tests (**a** and **c**). *N.S.*, not significant, $P > 0.05$; *, $P < 0.05$; **, $P < 0.005$. Source data are provided as a Source Data file.

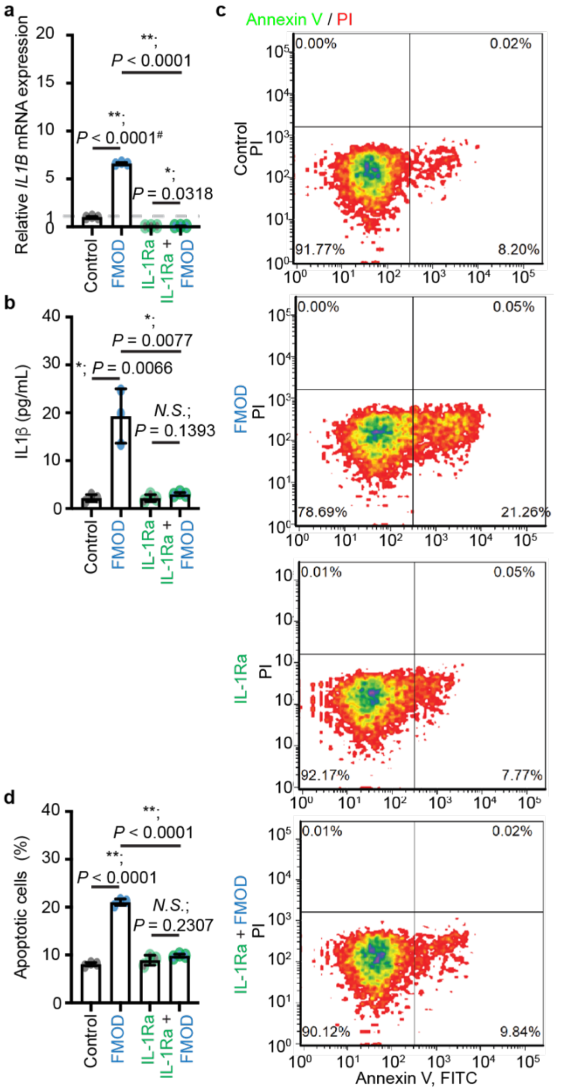

Supplementary Fig. 12. Interleukin-1 receptor antagonist (IL-1Ra) blocks fibromodulin (FMOD)-induced interleukin (IL)1β expression and apoptosis of BJ-myofibroblasts.

All cells were subjected to serum starvation prior to treatment. **a**. Expression of *IL1B* in BJ-myofibroblasts with or without IL-Ra treatment. Data were normalized to the *IL1B* level of the control. **b**. Active IL1β production in BJ-myofibroblasts with or without IL-Ra treatment. **c**. Representative plots of BJ-myofibroblasts with or without IL-Ra treatment stained with Annexin V-FITC and PI staining. **d**. Quantification of BJ-myofibroblast apoptosis from **c**. Data presented as mean ± s.d. overlaying all the data points. *N* = 3 biological replicates; *P* values were determined by two-tailed unpaired *t*-

tests (**a**, **b**, and **d**), except the one with marker # (**a**, in which data of the control group did not meet the criteria for normal distribution test and thus was treated as non-parametric) where a Mann-Whitney *U* test determined the *P* value. *N.S.*, significant, $P > 0.05$; *, $P < 0.05$, **; $P < 0.005$. Source data are provided as a Source Data file.

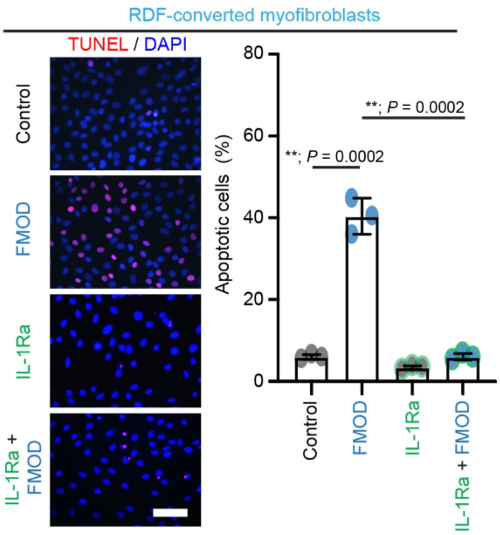

Supplementary Fig. 13. Interleukin-1 receptor antagonist (IL-1Ra) blocks fibromodulin (FMOD)-induced rat dermal fibroblast (RDF)-derived myofibroblast apoptosis.

The number of TUNEL-positively stained cells among 1,000 cells (determined by DAPI-stained nuclei) was counted under a microscope for each cell culture to determine the percentage of apoptotic cells. Data presented as mean ± s.d. overlaying all the data points. *N* = 3 biological replicates; *P* values were determined by two-tailed unpaired *t*-tests. **; $P < 0.005$. Source data are provided as a Source Data file.

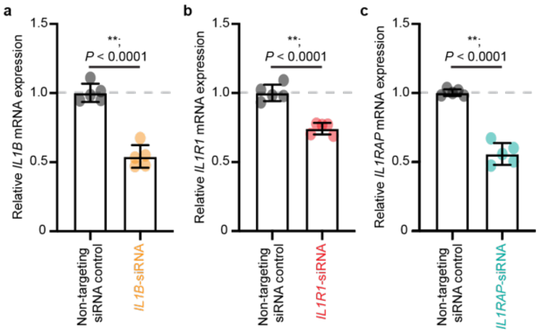

Supplementary Fig. 14. RNA interference (RNAi) successfully knocks down the expression of its target transcript in BJ-derived myofibroblasts (BJ-myofibroblasts).

All cells were subjected to serum starvation prior to treatment. **a**. Expression of *IL1B* in BJ myofibroblasts with *IL1B* knockdown. Data were normalized to the *IL1B* level of the BJ myofibroblasts transfected with the non-targeting siRNA control. **b**. Expression of *IL1R1* in BJ myofibroblasts with *IL1R1* knockdown. Data were normalized to the *IL1R1* level of the BJ myofibroblasts transfected with the non-targeting siRNA control. **c**. Expression of *IL1RAP* in BJ myofibroblasts with *IL1RAP* knockdown. Data were normalized to the *IL1RAP* level of the BJ myofibroblasts transfected with the non-targeting siRNA control. Data presented as mean ± s.d. overlaying all the data points. $N = 5$ biological replicates; P values were determined by two-tailed unpaired t-tests. **, $P < 0.005$. Source data are provided as a Source Data file.

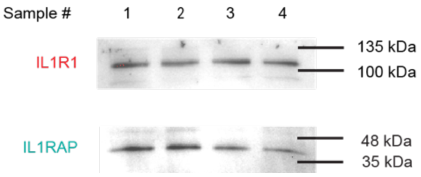

Supplementary Fig. 15. Western blotting confirms the staining pattern of interleukin 1 receptor type 1 (IL1R1)-interleukin-1 receptor accessory protein (IL1RAP) in the whole-membrane protein extracted from BJ-myofibroblasts. N = 4 biological replicates were tested for each antibody. Source data are provided as a Source Data file.

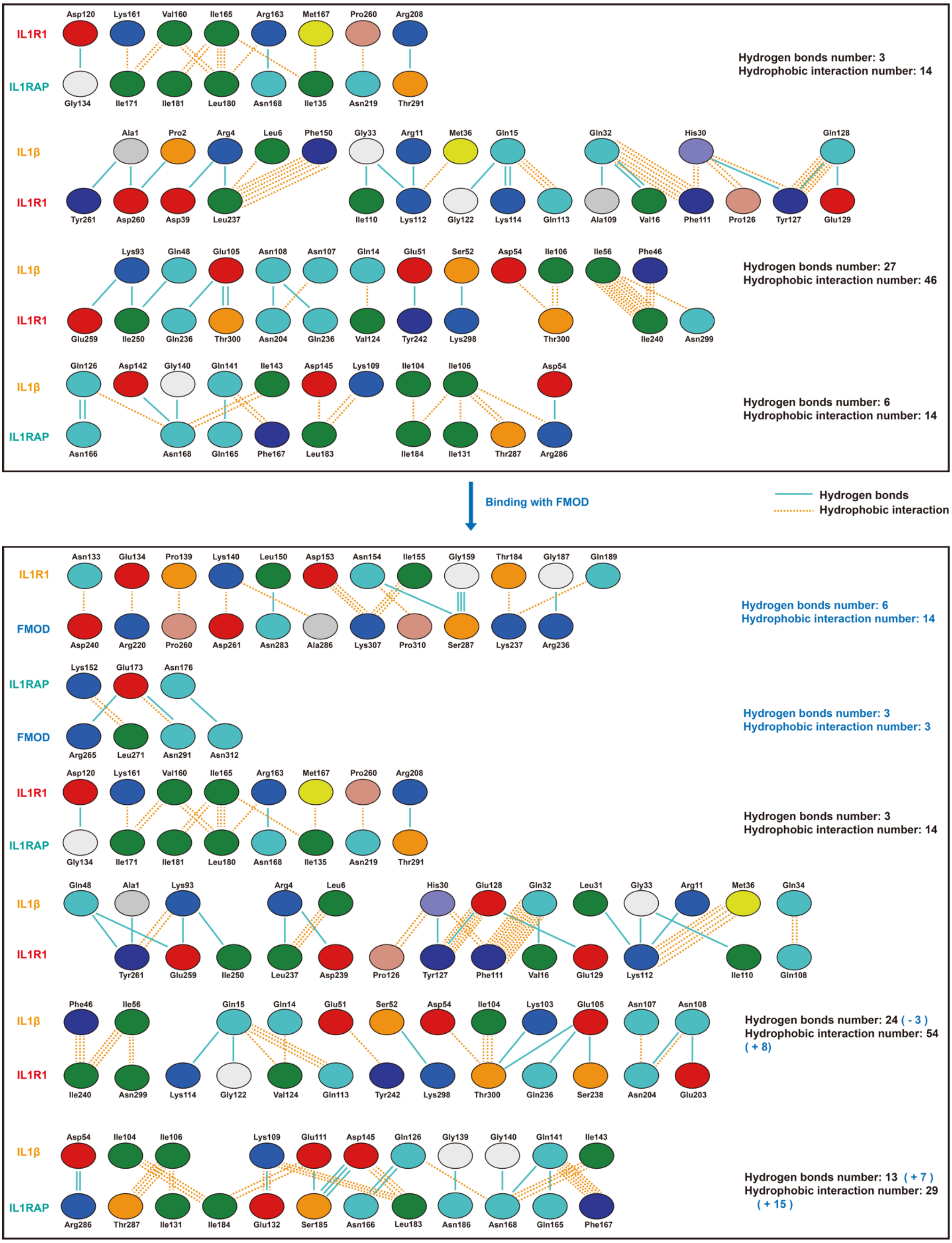

Supplementary Fig. 16. *In silico* analysis predicts the effects of fibromodulin (FMOD) on amino acid residue interactions among the interleukin (IL)1β-interleukin 1 receptor type 1 (IL1R1)-interleukin-1 receptor accessory protein

(IL1RAP) ternary complex components.

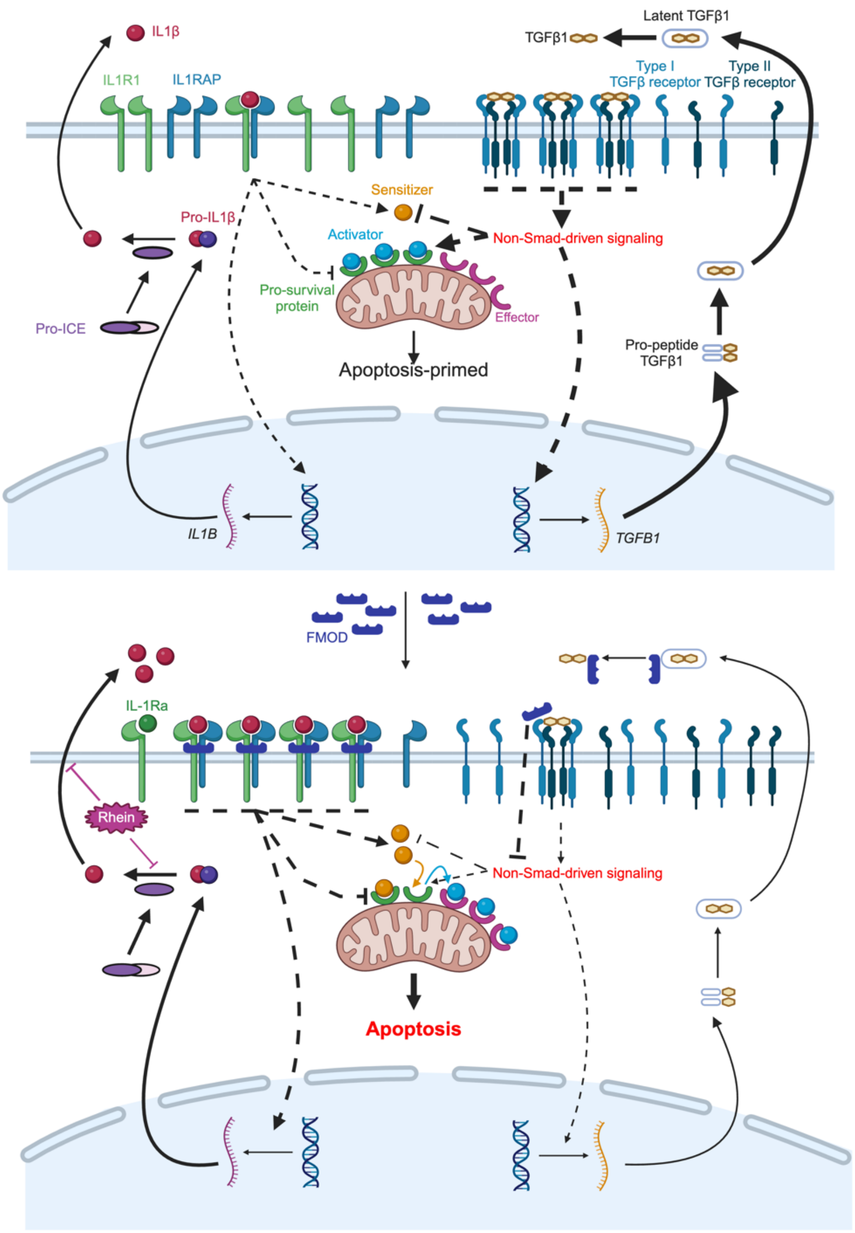

Supplementary Fig. 17. A schematic illustration of the potential mechanism by which FMOD induces myofibroblast apoptosis.

(**Upper**) TGFβ1, through its non-Smad-driven pathways, upregulates pro-survival BCL2 proteins and inhibits the sensitizer protein BAD. Consequently, TGFβ1 not only prevents myofibroblasts from undergoing apoptosis but also counteracts IL1β's promotion of myofibroblast apoptosis, which is associated with the downregulation of pro-survival BCL2 proteins and suppression of FAK signaling that inhibits the pro-apoptotic sensitizer BAD. (**Lower**) When exogenous FMOD is introduced, it

significantly reduces the binding energy of the IL1β-IL1R1 binary complex and the IL1β-IL1R1-IL1RAP ternary complex, facilitating the binding of IL1β with its cognate receptor IL1R1 and co-receptor IL1RAP. Thereby, FMOD enhances IL1β signal transduction, significantly amplifies the IL1β autocrine loop, and promotes targeted apoptosis of myofibroblasts. Consequently, IL-1Ra, which competes with IL1β for binding to IL1R1 without triggering any downstream signaling, and rhein, which hinders IL1β maturation and secretion, interrupt the IL1β autocrine loop and prevent myofibroblast apoptosis in response to FMOD treatment. Meanwhile, FMOD, through its N-terminus, can directly bind to TGFβ1 and its latent form, potentially sequestering TGFβ1 globally and preventing it from binding with its receptors to initiate downstream signal transduction, including pathways that allow myofibroblasts to escape apoptosis. FMOD also selectively dampens TGFβ1's non-Smad-driven signaling. As a result, FMOD can counteract TGFβ1's effect on preventing myofibroblast apoptosis. Given that the fragments of FMOD responsible for binding with TGFβ1 and the IL1β-IL1R1-IL1RAP ternary complex do not overlap, FMOD may simultaneously enhance IL1β signaling and weaken TGFβ1 non-Smad-driven pathways, thereby exhibiting a dual potency in inducing myofibroblast apoptosis. This figure was created with BioRender. Li. C. (2005) https://BioRender.com/u94o620.
