# Supplementary material for: Fibromodulin selectively accelerates myofibroblast apoptosis in cutaneous wounds by enhancing interleukin 1β signaling
Source: Nat Commun. 2025 Apr 12;16:3499. doi: 10.1038/s41467-025-58906-z (PMC11993684; doi:10.1038/s41467-025-58906-z)
Supplement: Supplementary file 2 — Reporting Summary [file 41467_2025_58906_MOESM2_ESM.pdf]

nature portfolio

Corresponding author(s): Kang Ting, Chia Soo, and Zhong Zheng

Last updated by author(s): Mar 15, 2025

Reporting Summary

Nature Portfolio wishes to improve the reproducibility of the work that we publish. This form provides structure for consistency and transparency in reporting. For further information on Nature Portfolio policies, see our Editorial Policies and the Editorial Policy Checklist.

Statistics

For all statistical analyses, confirm that the following items are present in the figure legend, table legend, main text, or Methods section.

n/a	Confirmed	
☐	☒	The exact sample size (*n*) for each experimental group/condition, given as a discrete number and unit of measurement
☐	☒	A statement on whether measurements were taken from distinct samples or whether the same sample was measured repeatedly
☐	☒	The statistical test(s) used AND whether they are one- or two-sided<br>*Only common tests should be described solely by name; describe more complex techniques in the Methods section.*
☒	☐	A description of all covariates tested
☐	☒	A description of any assumptions or corrections, such as tests of normality and adjustment for multiple comparisons
☐	☒	A full description of the statistical parameters including central tendency (e.g. means) or other basic estimates (e.g. regression coefficient) AND variation (e.g. standard deviation) or associated estimates of uncertainty (e.g. confidence intervals)
☐	☒	For null hypothesis testing, the test statistic (e.g. *F*, *t*, *r*) with confidence intervals, effect sizes, degrees of freedom and *P* value noted<br>*Give P values as exact values whenever suitable.*
☒	☐	For Bayesian analysis, information on the choice of priors and Markov chain Monte Carlo settings
☒	☐	For hierarchical and complex designs, identification of the appropriate level for tests and full reporting of outcomes
☒	☐	Estimates of effect sizes (e.g. Cohen's *d*, Pearson's *r*), indicating how they were calculated

Our web collection on statistics for biologists contains articles on many of the points above.

Software and code

Policy information about availability of computer code

Data collection	Olympus IX71 microscope coupled with a DP73 camera (Olympus, Cypress, CA); cellSens Standard 1.9 software (Olympus, Cypress, CA); Epoch Microplate Spectrophotometer (BioTek Instruments Inc., Winooski, VT); Gen5 software (version 2.04.11; BioTek Instruments Inc., Winooski, VT); QuantStudio3 system (Thermo Fisher Scientific); Flow cytometry (BDTM LSRII; BD Biosciences, San Jose, CA); Biacore 3000 instrument (Biacore AB, Uppsala, Sweden); CM5 sensor chips (GE Healthcare, Uppsala, Sweden); Protein Data Bank (PDB; Piscataway, NJ); and Leica TCS SP8 Confocal Laser Scanning Platform (Leica Biosystems, Wetzlar, Germany).
Data analysis	FCS Express 4 software (De Novo Software, Glendale, CA); Scrubber 2.0 (BioLogic Software Pty Ltd., Campbell, Australia); I-TASSER server (Iterative Threading Assembly Refinement; Yang Zhang Laboratory, University of Michigan, Ann Arbor, MI); PatchDock server (Institute of Molecular Medicine, Tel Aviv University, Tel Aviv, Israel); PDBePISA server (v1.52; EMBL-EBI; Cambridgeshire, UK); Arpeggio server (University of Cambridge, Cambridge, UK); Image J (version 1.52q; NIH); and Prism (version 8.2.1; GraphPad, San Diego, CA).

For manuscripts utilizing custom algorithms or software that are central to the research but not yet described in published literature, software must be made available to editors and reviewers. We strongly encourage code deposition in a community repository (e.g. GitHub). See the Nature Portfolio guidelines for submitting code & software for further information.

Data

Policy information about availability of data

All manuscripts must include a data availability statement. This statement should provide the following information, where applicable:

- Accession codes, unique identifiers, or web links for publicly available datasets
- A description of any restrictions on data availability
- For clinical datasets or third party data, please ensure that the statement adheres to our policy

All data supporting the findings of this study are included within the manuscript and Supplementary Information files. Source data for the main and supplementary figures are provided in a Source Data Excel file underlaying

Research involving human participants, their data, or biological material

Policy information about studies with human participants or human data. See also policy information about sex, gender (identity/presentation), and sexual orientation and race, ethnicity and racism.

Reporting on sex and gender	Donors' characteristics including sex are depicted Supplementary Table S1. This study did not involve gender or sexual orientation research.
Reporting on race, ethnicity, or other socially relevant groupings	Donors' characteristics including race and age are depicted Supplementary Table S1. This study did not involve ethnicity or other socially relevant grouping sstudy.
Population characteristics	This study did not involve population characteristics study.
Recruitment	No human participant recruitment was involved in the current study.
Ethics oversight	Human skin samples were procured by the National Disease Research Interchange (NDRI; Philadelphia, PA; the researcher code is DTIK2). According to the NDRI's policy, the signed consents/authorizations were kept on file at the tissue acquisition site without being disclosed to the investigators. Since the donated tissues are medically discarded wastes and no personally identifiable information is provided, UCLA institutional review boards (IRB) determined their usage was an exception for IRB review (Application number: IRB#19-000605).

Note that full information on the approval of the study protocol must also be provided in the manuscript.

Field-specific reporting

Please select the one below that is the best fit for your research. If you are not sure, read the appropriate sections before making your selection.

[x] Life sciences   [ ] Behavioural & social sciences   [ ] Ecological, evolutionary & environmental sciences

For a reference copy of the document with all sections, see nature.com/documents/nr-reporting-summary-flat.pdf

Life sciences study design

All studies must disclose on these points even when the disclosure is negative.

Sample size	Sample size calculations were not conducted in this study. For biochemical assays, the typical sample size was n = 3 (biologically independent experiments), consistent with standard practice in biochemical studies and our prior experience (e.g. Doring et al. Nat Cardiovasc Res, 2024; Burger-Kentischer et al. Circulation, 2002 ; Qi et al. J Clin Invest, 2009; Klasen et al., J. Immunol., 2014; Alampour-Rajabi et al., FASEB J., 2015; Kontos et al., Nat. Commun., 2020; Tas et al., Nat. Commun., 2022). Sample size for in vivo experiments were determined base on prior experience with the same situation, similar to our previous work (Zheng et al., Sig. Transduct. Target.. Ther., 2017; Jiang et al., J. Cell. Mol. Med.).
Data exclusions	No date was excluded from analyses.
Replication	The number of independently conducted experiments is indicated in the legends of the figures and supplementary figures throughout the manuscript, with all replications completed successfully.
Randomization	Animals were randomly assigned into treatment groups. For in vitro experiments, randomization was not used due to cell homogeneity and covariates were controlled by seeding all groups at the same cell densities and maintaining them in the same growth and media conditions except the intervention. Furthermore, different intervention groups were handled simultaneously whenever possible.
Blinding	All data were collected blindly.

Reporting for specific materials, systems and methods

We require information from authors about some types of materials, experimental systems and methods used in many studies. Here, indicate whether each material, system or method listed is relevant to your study. If you are not sure if a list item applies to your research, read the appropriate section before selecting a response.

Materials & experimental systems

n/a	Involved in the study
☐	☒ Antibodies
☐	☒ Eukaryotic cell lines
☒	☐ Palaeontology and archaeology
☐	☒ Animals and other organisms
☒	☐ Clinical data
☒	☐ Dual use research of concern
☒	☐ Plants

Methods

n/a	Involved in the study
☒	☐ ChIP-seq
☐	☒ Flow cytometry
☒	☐ MRI-based neuroimaging

Antibodies

Antibodies used	IHC: mouse anti--SMA antibody [4A4] (GTX60466, GeneTex, Irvine, CA; 1: 500 dilution), rabbit anti-cleaved caspase-3 antibody (#9661, Cell Signaling Technology,; 1:400 dilution), and horse anti-mouse/rabbit IgG antibody (H+L) (Universal), Biotinylated (BA-1400-2.1, Vector Laboratories; 1:50 dilution). IF: mouse anti-a-SMA antibody [4A4] (GTX60466, GeneTex; 1: 500 dilution), goat anti-mouse IgG(H+L) highly cross-adsorbed secondary antibody, Alexa FluorTM 594 (A-11032, Thermal Fisher Scientific; 1:400 dilution), and phalloidin-iFluor 488 reagent (ab176753, Abcam; 1:800 dilution). Western blotting: goat anti-human IL-1 beta/IL-1F2 antibody (AF-201-NA, R&D systems; 0.1 ug/mL), rabbit anti-glyceraldehyde-3-phosphate dehydrogenase (GAPDH) antibody (PA1-987, Thermo Fisher Scientific; 1:3000 dilution), rabbit anti-fibromodulin antibody (H50) (sc-33772, Santa Cruz Biotechnology; 1:500 dilution), rabbit anti-IL1 receptor I/IL-1R-1 antibody (ab154524; Abcam; 1:1000 dilution), mouse-anti IL-1RAcP (D5) (sc-376872, Santa Cruz Biotechnology; 1:100 dilution), donkey anti-goat IgG (H+L) secondary antibody, HRP (A15999, Thermo Fisher Scientific; 1:5000 dilution), donkey anti-rabbit IgG (H+L) secondary antibody, HRP (A16023, Thermo Fisher Scientific; 1:5000 dilution), and donkey anti-mouse IgG (H+L) cross-adsorbed secondary antibody, HRP (SA1-100, Thermo Fisher Scientific; 1: 2000 dilution). FACS: Mouse anti--SMA antibody [4A4] (GTX60466, GeneTex, Irvine, CA; 1: 250 dilution) and goat anti-mouse IgG(H+L) highly cross-adsorbed secondary antibody, Alexa FluorTM 405 (A-31553, Thermal Fisher Scientific; 2 ug/mL). PLA: rabbit anti-IL1 receptor I/IL-1R-1 antibody (ab154524, Abcam; 1:200 dilution) and goat anti-human IL-1 beta/IL-1F2 antibody, AF-201-NA; R&D systems; 5 ug/mL) or mouse-anti IL-1RAcP (D5) (sc-376872, Santa Cruz Biotechnology; 1:50 dilution).
Validation	All antibodies are validated by the vendors and have been used according to the manufacturers instructions.

Eukaryotic cell lines

Policy information about cell lines and Sex and Gender in Research

Cell line source(s)	BJ cells were obtained from ATCC.
Authentication	BJ cells were authenticated by Laragen (Culver City CA).
Mycoplasma contamination	None of these primary cells were mycoplasma contamination.
Commonly misidentified lines (See ICLAC register)	No commonly misidentified lines were used in this study.

Animals and other research organisms

Policy information about studies involving animals; ARRIVE guidelines recommended for reporting animal research, and Sex and Gender in Research

Laboratory animals	3-month old 129/sv wild-type and Fmod-/- (B6;129-FMOD<tm1Aol>/SooJ, which is available at the Jackson Laboratory Repository with the JAX Stock No. 037010) mice; adult Sprague-Dawley rats (weighting ~300g); 20-kg red Duroc pigs (Pork Power Farms, Turlock, CA)
Wild animals	This study did not involved wild animals
Reporting on sex	Only male rodents and female pigs were used in this study.
Field-collected samples	This study did not involved field-collected samples.
Ethics oversight	All animal surgeries were performed following the United States National Institute of Health (NIH) Guide for the Care and Use of Laboratory Animals set forth under the institutionally approved protocols provided by the Chancellor's Animal Research Committee at the University of California, Los Angeles (UCLA; protocol number: 2000-058 and 2008-016).

Note that full information on the approval of the study protocol must also be provided in the manuscript.

Plants

Seed stocks: N/A

Novel plant genotypes: N/A

Authentication: N/A

Flow Cytometry

Plots

Confirm that:

- [x] The axis labels state the marker and fluorochrome used (e.g. CD4-FITC).
- [x] The axis scales are clearly visible. Include numbers along axes only for bottom left plot of group (a 'group' is an analysis of identical markers).
- [x] All plots are contour plots with outliers or pseudocolor plots.
- [x] A numerical value for number of cells or percentage (with statistics) is provided.

Methodology

Sample preparation: Generally, cell apoptosis was quantified by using the Dead Cell Apoptosis Kit with Annexin V-fluorescein isothiocyanate (FITC) and propidium iodide (PI) (Thermo Fisher Scientific) according to the manufacturer's instructions. Briefly, the testing cells were harvested 48 h post-treatment and washed with cold PBS. Cell density was adjusted to 1 × 10^5 cells/ml in 1× annexin-binding buffer and stained with Annexin V-FITC and PI for 15 min at room temperature. The apoptosis rate of staining cells was determined using Flow cytometry (BDTM LSRII; BD Biosciences, San Jose, CA) with excitation at 488 nm and emission at 523 nm.
Since an enhanced green fluorescent protein (EGFP) expression cassette was introduced into the IL1B-knockout BJ fibroblasts by the lentivirus vector YKO-LV001-dual-gRNA, Annexin V allophycocyanin (APC) conjugate (Thermo Fisher Scientific) and DAPI were used instead to assess the apoptosis of IL1B-knockout myofibroblasts. The flow cytometry was conducted with excitation at 633 nm and emission at 660 nm.

Instrument: Flow cytometry (BDTM LSRII; BD Biosciences, San Jose, CA)

Software: FCS Express 4 software (De Novo Software, Glendale, CA)

Cell population abundance: Cell density was adjusted to 1 × 10^5 cells/mL

Gating strategy: A standard protocol was employed to set the gating by using cells treated with staurosporine (50 nM, 2 h; MilliporeSigma) treatment as the apoptosis control71, and cells treated with LPS (100 ng/mL) for 3 h followed by 1-h co-treatment with LPS and nigericin (5m; MilliporeSigma) as the pyroptosis control72. After using unstained controls to identify autofluorescence, single-stained controls for PI and Annexin V-FITC were used to set the compensation matrix, and the boundary between the apoptotic and pyroptotic cells was confirmed by comparing the double-stained controls following the instruction of UCLA Broad Stem Cell Research Center Flow Cytometry Core Resource. Using this Annexin V/PI apoptotic detecting method, flow cytometry categorizes the Annexin V-/PI+ population as necrotic cells, the Annexin V+/PI+ population as pyroptotic cells, and the Annexin V+/PI- population as apoptotic cells.
When cells were stained with Annexin V allophycocyanin (APC) conjugate (Thermo Fisher Scientific) and DAPI, a similar gating setting procedure described above has also been used for the DAPI and Annexin V-APC staining. Notably, using the resulting DAPI/APC gating, control- and FMOD-treated wild-type BJ-myofibroblasts without IL1B-knockout exhibited comparable percentages of apoptotic cells as using PI/FITC gating. Thus, this DAPI/APC gating was used to analyze IL1B-knockout BJ-myofibroblasts in the same experimental setting to ensure accurate gating and rigorous and reliable comparison.

- [ ] Tick this box to confirm that a figure exemplifying the gating strategy is provided in the Supplementary Information.
